# Development of probiotics beverage using cereal enzymatic hydrolysate fermented with *Limosilactobacillus reuteri*

**DOI:** 10.1002/fsn3.2913

**Published:** 2022-05-03

**Authors:** Zhoujie Yang, Xiaoli Zhu, Anyan Wen, Likang Qin

**Affiliations:** ^1^ Key Laboratory of Plant Resource Conservation and Germplasm Innovation in Mountainous Region (Ministry of Education) College of Life Sciences/Institute of Agro‐bioengineering Guizhou University Guiyang Guizhou Province China; ^2^ School of Liquor and Food Engineering Guizhou University Guiyang Guizhou Province China

**Keywords:** cereal, coix seed, *Limosilactobacillus reuteri*, volatile flavor compounds

## Abstract

Although most probiotic products are milk based, lactose intolerance and vegetarianism inspired the idea of developing nondairy probiotic products. In this study, probiotic beverages were produced from four enzymatically hydrolyzed cereal substrates (coix seed, quinoa, millet, and brown rice) and fermented by *Limosilactobacillus reuteri*. Fermentation parameters, including pH, titratable acidity, viable count, organic acids, and volatile components were determined. Results showed that the pH values decreased and titratable acidity increased with the fermentation process (*p* < .05). Although the final pH in all samples was below 4.0, the growth of *L. reuteri* was not significantly inhibited by low pH. The number of viable bacteria (12.96 log CFU/ml) in coix seed substrate was significantly higher than that in other samples after the fermentation for 24 h (*p* < .05). Lactic acid and acetic acid were the main organic acids after fermentation and the highest in quinoa (lactic acid: 7.58 mg/ml; acetic acid: 2.23 mg/ml). The flavor analysis indicated that there were differences in the flavor components of different cereal beverages. Forty‐nine volatile compounds were identified in four beverages, including acids, alcohols, aldehydes, ketones, and esters. The results of the electronic tongue showed that the umami taste of the fermented coix seed was better than that of other samples, displaying the more pleasant taste characteristics. In conclusion, it is feasible to prepare probiotic symbiotic cereal beverage with *L*. *reuteri* as starter culture. This study provides a reference for the development of nondairy probiotic products.

## Graphical abstract

This study was aimed to develop probiotic beverage by using *Limosilactobacillus reuteri* and enzymatically hydrolyzed cereals. Furthermore, the pH, titratable acidity, viable count, organic acids, and volatile components were investigated.

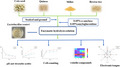

## 1 INTRODUCTION

Probiotics have become a hot topic in the fields of food and medicine due to their beneficial effects on hosts. Probiotics play a vital role in maintaining the intestinal microecology and preventing chronic diseases (Kamada et al., 2013; Sullivan et al., 2001). At present, fermented dairy products are still the predominant carriers of probiotics (Gupta & Bajaj, 2017). However, the increasing concerns on lactose intolerance, milk protein allergy, high cholesterol content, and high contents of saturated fatty acids of dairy‐based foods limit the population of consumers (Vijaya Kumar et al., 2015). Therefore, nondairy probiotics have received great attention to meet more food consumption demand. Except for fermented dairy products, probiotics have also been widely used to ferment fruits (Chen et al., 2019), vegetables (Tomita et al., 2021), and cereals ( Zhao, Wu, et al., 2021).

For a long time, cereals have been a source of energy and nutrients for human beings. Proteins, carbohydrates, fiber, and minerals in cereals are important sources of the human nutritional needs (Blandino et al., 2003). However, the application scope of cereals in other products is largely limited by their poor sensory quality. Therefore, a number of processing methods are employed to improve the nutritional properties of cereals, including germination, milling, and fermentation. Fermentation is one of the oldest and most economical methods of producing and preserving foods. Probiotics fermentation of cereals may enhance the bioavailability, digestibility, and organoleptic properties (Enujiugha & Badejo, 2017). In addition, the nutrients in cereals can promote the growth of probiotics (Arora et al., 2010). Many studies had shown that cereals were good substrates for the growth of probiotics (Casarotti et al., 2014; Matejčeková et al., 2017; Russo et al., 2016a). The presence of prebiotics in cereals can promote the growth of probiotics (Noori et al., 2017).

Flavor is one of the important factors that affect the sensory quality of products and determines the acceptability and preference of products (Pan et al., 2014). Fermentation with lactic acid bacteria (LAB) is considered to be an effective way to improve food flavor (Bartkiene et al., 2015; Nedele et al., 2021). Lactic acid bacteria have complex enzyme systems and produce a number of metabolites to provide different flavor characteristics of products (Gänzle et al., 2009). In recent years, LAB have been widely used in various cereal products to improve their nutrition and sensory (Luana et al., 2014; Soukoulis et al., 2014). However, the volatile composition and sensory profile of lactic acid fermentation with different cereals were different even though the same LAB strain was used for fermentation (Nsogning Dongmo et al., 2017).

Probiotic products based on cereals are an important development direction of functional foods. To date, a variety of probiotics have been used in the development of functional cereal products, including *Lactobacillus plantarum* (Yin et al., 2020), *Lactobacillus acidophilus* (Mis Solval et al., 2019), *Limosilactobacillus reuteri* (Pallin et al., 2016), and *Lactobacillus casei* (Li et al., 2018). *Limosilactobacillus reuteri* widely inhabit the gastrointestinal tract of mammals and its probiotic effects have been well documented (Emara et al., 2014; Sung et al., 2018). Additionally, *L. reuteri* has been found in sourdoughs made from cereals (De Vuyst et al., 2014). However, there had been no reports on the development of probiotic beverage fermented with *L. reuteri* based on enzymatically hydrolyzed cereals. Therefore, this study was aimed to develop probiotic beverage by using *L. reuteri* and enzymatically hydrolyzed cereals. The pH, titratable acidity, viable count, organic acids, and volatile components were investigated. The study provides the new insights into the development of functional cereal products with highly viable probiotics.

## 2 MATERIALS AND METHODS

### 2.1 Materials

Four cereals were selected and used in the fermentation process with *L*. *reuteri*. Coix seed was obtained from Guizhou Renxin Agriculture Development Co., Ltd. (Guizhou, China). Quinoa was procured from Qinghai Xinlvkang Food Co., Ltd. (Qinghai, China). Millet and brown rice were purchased from a local market.

### 2.2 Microorganisms

The probiotic microorganism used in this study was *Limosilactobacillus reuteri* BNCC186563 purchased from Bena Culture Collection (Suzhou, China). The strain was subcultured three times for 24 h at 37°C in De Man, Rogosa, and Sharpe broth (MRS; Shanghai Bio‐way Technology Co., Ltd., Shanghai, China) before the experiment. The starter culture was cultured and inoculated for 16 h at 37°C in MRS broth. Then, it was centrifuged at 3000 rpm for 5 min, washed in 0.9% saline, and diluted with 0.9% saline to obtain a probiotic culture with a cell concentration about 10^9^ CFU/ml, which served as the inoculum for the cereal fermentation substrates.

### 2.3 Preparation and fermentation of cereal substrates

After adding deionized water, coix seed, quinoa, millet, and brown rice were soaked at 4°C for 12 h. Water was drained and distilled water was added into cereals to a ratio of 1:10 (seed to water) and ground in an electric grinder. The suspension was gelatinized at 90°C for 30 min and then cooled to room temperature. Then, 0.05% α‐amylase (3700 U/g, Beijing Solarbio Science & Technology Co., Ltd., Beijing, China) and 0.05% amyloglucosidase (100,000 U/g, Beijing Solarbio Science & Technology Co., Ltd., Beijing, China) were added into the suspension. Enzymolysis was performed at 65°C for 30 min. Finally, the suspension was filtered through 120‐mesh cloth. The filtered suspension was steamed at 121°C for 10 min and cooled to 37°C. All samples were inoculated with 5% (V/V) probiotic cultures. The fermentations were performed in 250 ml Erlenmeyer flask, including 100 ml of the substrates, and fermented for 24 h at 37°C in an anaerobic incubator (10% H_2_ + 10% CO_2_ + 80% N_2_, Gene Science AG300, USA).

### 2.4 Determination of pH and titratable acidity

The pH of the samples was evaluated every 6 h using a digital pH meter (Testo, Germany). Titratable acidity (TA) measured by 0.1 N sodium hydroxide solution and 1% ethanol solution of phenolphthalein used an indicator, the results expressed in % lactic acid (Wang et al., 2019).

### 2.5 Cell counting

The quantity of viable cells of *L*. *reuteri* was determined by counting the colony forming units (CFU) on MRS agar (Hati et al., 2018). First, 1.0 ml of sample was added into 9 ml of sterile saline and then serially diluted. The dilution was used for microbial enumeration with MRS agar plates. These plates were cultured anaerobically (10% H_2_ + 10% CO_2_ + 80% N_2_, Gene Science AG300, USA) for 48 h at 37°C. Then the colonies formed were counted and expressed as log CFU/ml.

### 2.6 HPLC analysis of organic acids

Organic acids (lactic acid and acetic acid) were analyzed by high‐performance liquid chromatography equipped with an ultraviolet detector (Agilent, USA). The supernatant was centrifuged at 10,000 *g* for 10 min and then filtered through a Millex‐HA 0.22‐µm pore size filter. The mobile phase was 0.02 M NaH_2_PO_4_ (pH = 2.7) and the flow rate of 0.6 ml/min. The isocratic elution procedure was adopted. The column temperature was set at 35°C and the detection wavelength was 210 nm. All organic acids were determined with different concentrations of standards (Pereira et al., 2017).

### 2.7 Determination of volatile compounds by GC‐MS

Gas chromatography‐mass spectrometry (GC‐MS) was used to analyze the volatile compounds from fermentation products. After the fermentation process, the samples were centrifuged at 8000 *g* for 10 min at 4°C and the supernatant was taken for analysis. An appropriate volume of the supernatant was placed in a 20‐ml headspace bottle, the 50/30 µm UMCAR /PDMS/DVB extraction head was inserted into the headspace part of the sample bottle for 30 min adsorption at 60°C. Then the extraction head was taken out and inserted into the gas chromatography inlet for 3 min desorption at 250°C.

The GC‐MS system (Pegasus BT [LECO, USA] Agilent Ltd., USA) was equipped with a Zebron ZB‐5MSi capillary column (5% phenyl and 95% dimethylpolysiloxane; 30 m × 0.25 mm × 0.25 μm; Phenomenex, USA). Operating conditions were set as follows. The initial temperature at 40°C was kept for 3 min. The temperature was increased to 230°C at a rate of 10°C/min and maintained for 5 min. Helium was used as the carrier gas and its flow rate was 1.0 ml/min. The temperatures of ion source, transfer line, and quadrupoles were set at 250, 250, and 200°C, respectively. The electron impact energy was 70 eV and the mass range was 29–500 amu.

The volatile compounds were identified by matching the mass spectra of the respective compounds to the database (Wiley275/NIST2008) and retention index (RI). Retention indices were determined by analysis of C6–C26 alkane standards under the same chromatographic conditions. The area normalization method was used to determine the relative percentage of the volatile compounds.

### 2.8 Electronic tongue analysis

The taste analysis of samples was performed with the taste sensing system SA402B (Intelligent Sensor Technology Inc., Japan). The system was composed of six test sensors, which were indicated as AAE (umami), CA0 (sourness), CT0 (saltiness), C00 (bitterness), AE1 (astringency), and GL1 (sweetness). Before the test, the system was checked to ensure the stability and reliability of the obtained data.

During the test, samples were added into sample cups. Then sensors were cleaned three times and each cleaning time was 90, 120, and 120 s, respectively. After cleaning, sensors were balanced together with samples for 30 s before measurement. Each sample was cycled four times. The data of the first cycle were discarded and the remaining three cycles were analyzed. After each test, sensors were cleaned automatically (Tian et al., 2020).

### 2.9 Statistical analysis

All experiments were performed in triplicate and expressed as mean values ± standard deviation. Data analyses were conducted with SPSS Version 19.0 software package for Windows. The difference was considered to be statistically significant at *p* < .05.

## 3 RESULTS AND DISCUSSION

### 3.1 pH and titratable acidity

**FIGURE 1:**
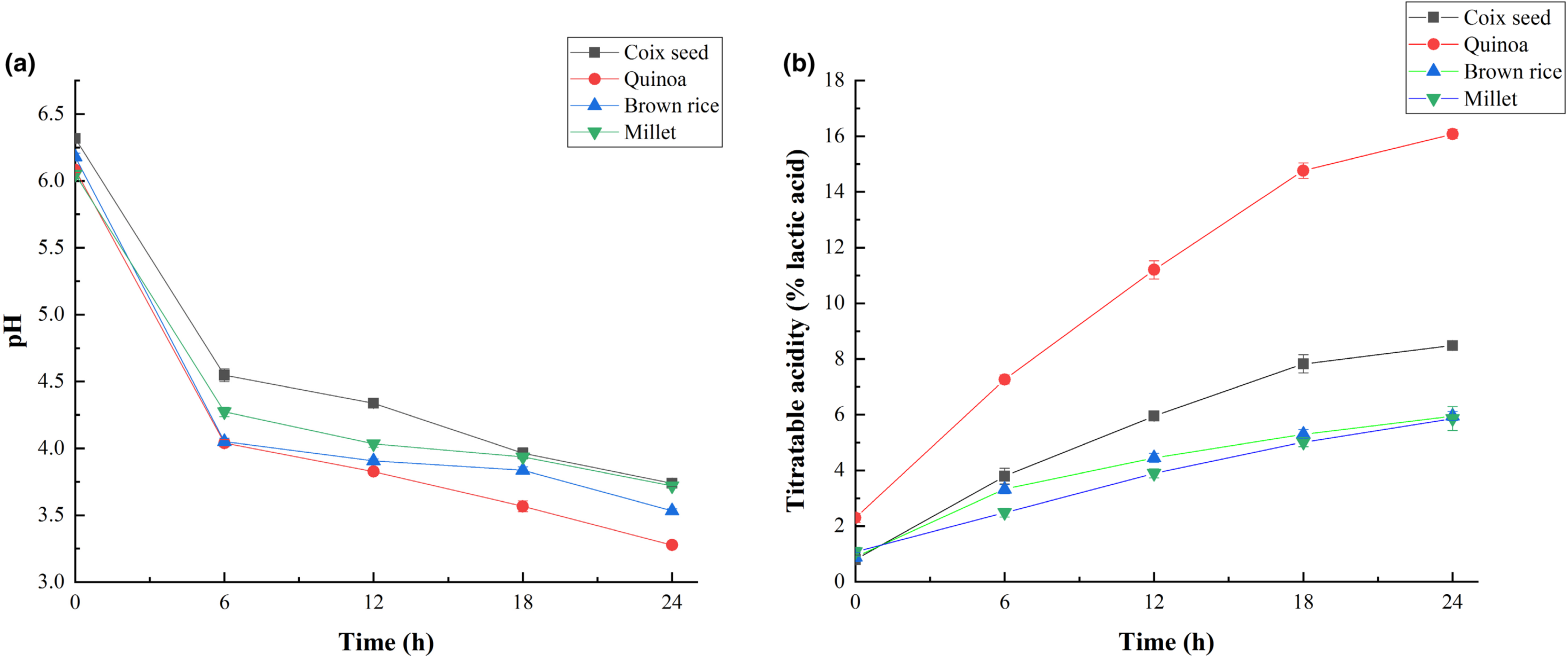
Change in pH (a) and titratable acidity (b) of enzymatically hydrolyzed cereal substrates fermented by *Limosilactobacillus reuteri*

*Limosilactobacillus reuteri* is an obligate heterozygous fermentation strain and can produce lactic acid, acetic acid, and carbon dioxide (Ichinose et al., 2020). Figure 1a shows the pH change during the fermentation process. At the beginning of fermentation, the pH values of coix seed, quinoa, brown rice, and millet substrates were 6.32 ± 0.02, 6.06 ± 0.02, 6.16 ± 0.03, and 6.08 ± 0.03, respectively. After 24‐h fermentation, the pH values of all the substrates were decreased to 3.74 ± 0.03 (coix seed), 3.28 ± 0.02 (quinoa), 3.53 ± 0.02 (brown rice), and 3.72 ± 0.01 (millet), respectively. The pH values of quinoa were decreased most significantly in all the substrates (*p* < .05). In the early stage of fermentation (within 6 h), the pH values decreased rapidly, which might be related to the accumulation of organic acids (Kedia et al., 2007). In addition, the carbon dioxide produced by fermentation might also lead to decrease in pH. The titratable acidity of samples increased during the fermentation process (Figure 1b). Among all the substrates, quinoa showed the most significant increase in titratable acidity (*p* < 0.05). The increasing trend of acidity was basically consistent with the decreasing trend of pH.

### 3.2 Growth profile of *Limosilactobacillus reuteri* in fermentation medium

**FIGURE 2:**
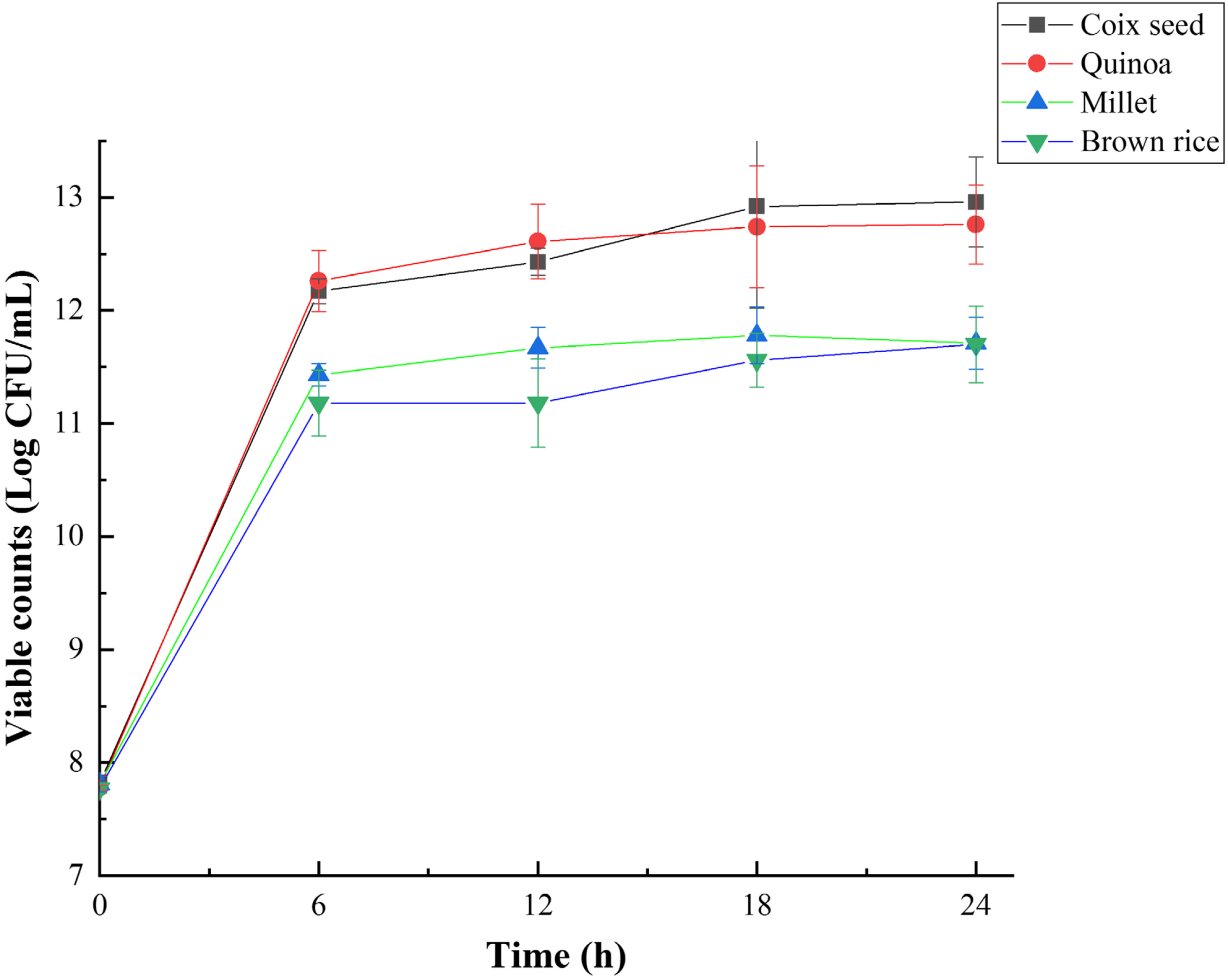
Growth profile of *Limosilactobacillus reuteri* in enzymatically hydrolyzed cereal substrates during fermentation. Values are presented as mean ± standard deviation (*n* = 3)

Cereals provide rich nutrition for the growth of *L*. *reuteri*. In this study, the growth state of *L*. *reuteri* in cereal substrates within 24 h was analyzed (Figure 2). At the first 6 h of fermentation, *L*. *reuteri* grew rapidly in cereal substrates. At the end of fermentation, the quantities of viable cells reached 12.96 ± 0.40 log CFU/ml in coix seed, which was higher than the results reported by Helland et al. (2004). The quantities of viable cells in quinoa, millet, and brown rice were 12.76 ± 0.35, 11.71 ± 0.23, and 11.70 ± 0.34 log CFU/ml, respectively. The number of viable bacteria in coix seed was the highest compared with the other three cereals. Previous study had reported that cereal extracts can promote the growth of probiotics (Noori et al., 2017). Nakkarach and Withayagiat (2018) found that riceberry malt extract could significantly promote the growth of *Lactobacillus johnsonii* KUN119‐2, and the number of viable bacteria exceeded 11 log CFU/ml after 24 h of fermentation, similar results had been observed in our research. On the other hand, an appropriate pH is an important factor for the rapid growth of *L. reuteri*. It is reported that the optimal pH for the growth of *L. reuteri* is 4.5–6.8 (Otto Kandler, 1980). During the first 12 h of fermentation, the pH values of all samples were within the pH range suitable for *L. reuteri* growth. In addition, the acid buffering capacity of growth medium may also affect the growth of *L. reuteri*. Giger‐Reverdin et al. (2002) pointed the differences in the buffering capacity of different cereals against acids. Growth media with higher buffering capacity could better support bacterium growth to resist the inhibition of bacterium growth by low pH (Pallin et al., 2016). This difference may also exist between the cereals used in this study. This may explain why the final pH in different cereal substrates was similar, but the final bacterial density in different cereal substrates was significantly different. Amino acids in the medium may also affect the cell biomass of *L. reuteri*. It has been suggested that *L. reuteri* requires sufficient amino acids for optimal growth, such as methionine, glutamate, leucine, valine, and alanine (Mis Solval et al., 2019a). Previously, the analysis results of free amino acids in raw materials showed that coix seed and quinoa were higher than millet and brown rice in both kinds and quantities of free amino acids, while coix seed and quinoa were close to each other (data not shown).

### 3.3 Organic acids

Organic acids are important metabolites of lactic acid bacteria during the fermentation process and contribute to the decrease in pH and the inhibition on the growth of potential pathogens (Greifová et al., 2017). Lactic acid and acetic acid are the main organic acids in the fermentation of *L*. *reuteri*. Thus, we analyzed the contents of lactic acid and acetic acid in four substrates during the fermentation process (Table 1). Before fermentation, lactic acid or acetic acid was not detected in all substrates. After 24 h of fermentation, the contents of lactic acid in different substrates were significantly different (*p <* .05). The contents of organic acids rapidly increased when *L*. *reuteri* entered the logarithmic growth phase. The content of lactic acid in quinoa was the highest (7.58 ± 0.03 mg/ml) and the content of acetic acid (2.23 ± 0.02 mg/ml) in quinoa was also significantly higher than other substrates (*p <* .05), which might be related to the fermentable sugars in quinoa. It was reported that quinoa starches were more readily hydrolyzed to glucose without pepsin preincubation step (Sathaporn Srichuwong & Lamothe, 2017), thus contributing more to the accumulation of organic acid in quinoa. The contents of lactic acid and acetic acid in coix seed were 3.48 ± 0.02 and 0.31 ± 0.01 mg/ml, respectively. The lactic acid and acetic acid of millet fermented by *L*. *reuteri* were 2.13 ± 0.03 and 0.31 ± 0.02 mg/ml, respectively. The contents of lactic acid and acetic acid in brown rice substrates were 2.96 ± 0.02 and 0.25 ± 0.03 mg/ml, respectively. Lactic acid is the main flavor source in many fermented foods (Batista et al., 2017). Its unique sourness can increase the taste of foods. In addition, a proper concentration of acetic acid can increase the sourness of foods, but a too high concentration of acetic acid is irritant and not conducive to the taste of foods. Except quinoa, the other three substrates maintained a low concentration of acetic acid.

**TABLE 1:** Organic acid concentrations (mg/ml) in enzymatically hydrolyzed cereal substrates fermented by *Limosilactobacillus reuteri*

Organic acid	Sample	Time points during fermentation (h)				
		0	6	12	18	24
Lactic acid	Coix seed	–	1.74 ± 0.01^c^	2.32 ± 0.01^c^	3.24 ± 0.02^b^	3.48 ± 0.02^b^
	Quinoa	–	5.28 ± 0.02^a^	6.20 ± 0.01^a^	7.52 ± 0.03^a^	7.58 ± 0.03^a^
	Millet	–	0.61 ± 0.03^d^	1.33 ± 0.03^d^	2.04 ± 0.06^d^	2.13 ± 0.03^d^
	Brown rice	–	1.83 ± 0.01^b^	2.61 ± 0.03^b^	2.91 ± 0.02^c^	2.96 ± 0.02^c^
Acetic acid	Coix seed	–	0.18 ± 0.01^B^	0.28 ± 0.02^B^	0.29 ± 0.01^B^	0.31 ± 0.01^B^
	Quinoa	–	0.43 ± 0.02^A^	1.72 ± 0.02^A^	1.76 ± 0.01^A^	2.23 ± 0.02^A^
	Millet	–	0.19 ± 0.01^B^	0.23 ± 0.03^C^	0.28 ± 0.02^B^	0.31 ± 0.02^B^
	Brown rice	–	0.19 ± 0.01^B^	0.23 ± 0.01^C^	0.24 ± 0.02^C^	0.25 ± 0.03^C^

Note

“–” means not detected.

Results are expressed as mean ± standard deviation (*n* = 3). Different letters in the same column indicate significant differences (*p* < .05).

### 3.4 Volatile compound profiles determined with HS‐SPME/GC‐MS

**FIGURE 3:**
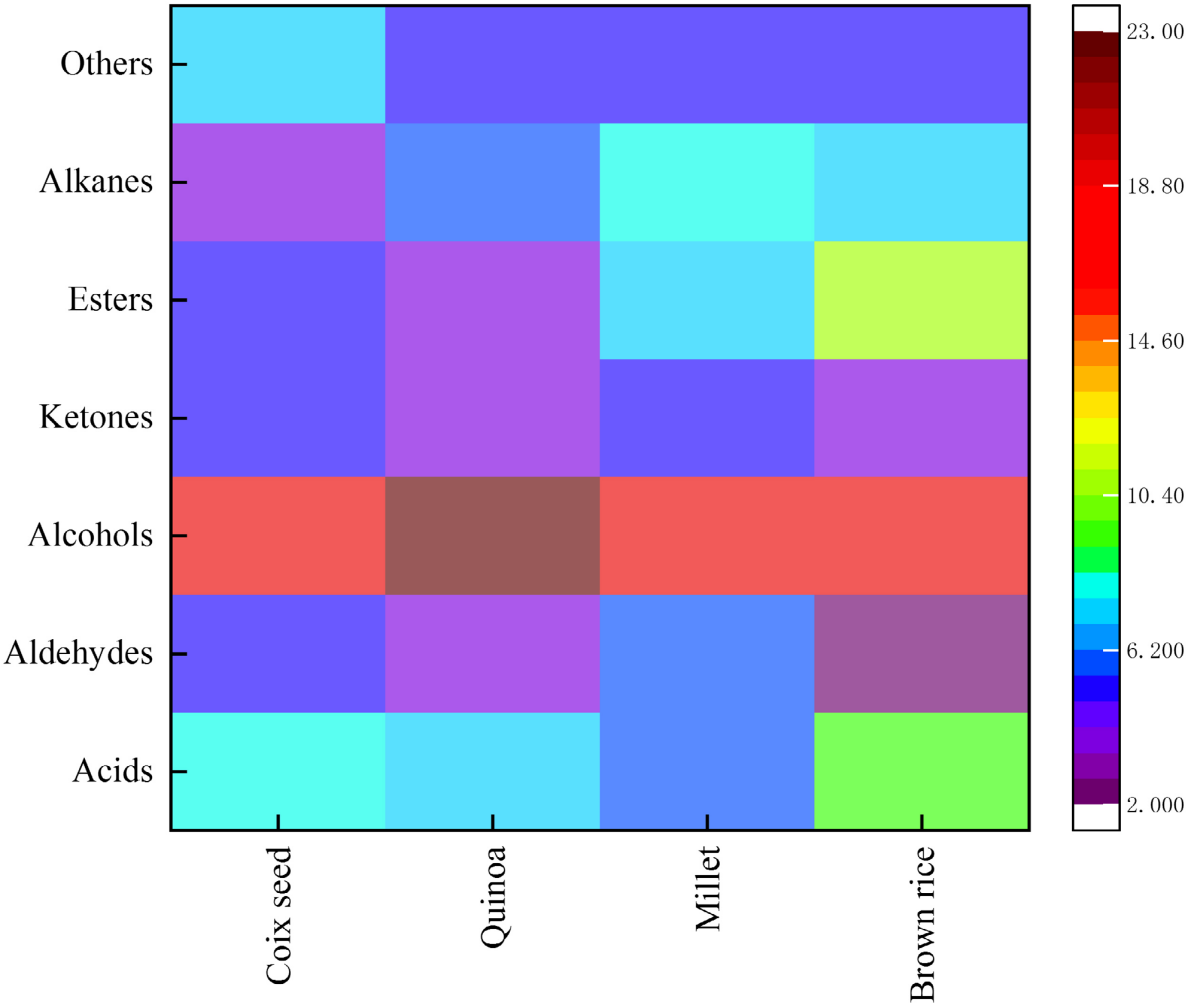
Comparison of volatile flavor compounds in different enzymatically hydrolyzed cereal substrates fermented by *Limosilactobacillus reuteri*

In this study, the flavor‐related compounds in four fermentation substrates were isolated by solid‐phase microextraction and identified by gas chromatography‐mass spectrometry (GC‐MS). The main aroma compounds were acids, alcohols, aldehydes, ketones, and esters after fermentation (Figure 3). Different substrates had different flavor components after the fermentation process (Table 2).

**TABLE 2:** Analyzed the volatile flavor compounds in different fermentation samples by SPME‐GC‐MS

	Compounds	RI	RT (S)	Relative area (%)			
				Coix seed	Quinoa	Millet	Brown rice
Acids	Acetic acid	1449.30	754.27	19.17 ± 0.06	26.49 ± 0.08	5.68 ± 0.04	9.99 ± 0.12
	Hexanoic acid	1838.10	1027.61	1.49 ± 0.02	1.27 ± 0.02	7.81 ± 0.03	1.58 ± 0.01
	Octanoic acid	2051.80	1156.88	0.49 ± 0.01	0.90 ± 0.02	1.29 ± 0.04	1.53 ± 0.06
	Nonanoic acid	2158.10	1216.86	0.18 ± 0.01	1.10 ± 0.02	0.49 ± 0.01	1.66 ± 0.03
	Decanoic acid	2263.90	1274.15	−	1.75 ± 0.01	0.04 ± 0.01	3.06 ± 0.02
Alcohols	Ethanol	935.00	290.93	−	22.75 ± 0.13	−	0.02 ± 0.01
	3‐Methyl−1‐butanol	1208.20	547.13	0.21 ± 0.02	0.38 ± 0.03	0.07 ± 0.01	−
	1‐Pentanol	1248.50	583.92	0.70 ± 0.02	0.48 ± 0.01	2.40 ± 0.06	0.60 ± 0.03
	2‐Heptanol	1311.80	640.96	1.85 ± 0.05	1.03 ± 0.03	4.17 ± 0.07	0.82 ± 0.01
	1‐Hexanol	1346.50	670.27	18.25 ± 0.12	5.27 ± 0.02	32.94 ± 0.23	7.03 ± 0.04
	2‐Octanol	1409.20	722.69	−	3.87 ± 0.06	7.91 ± 0.02	6.14 ± 0.03
	(R)−2‐Octanol	1409.30	722.77	7.11 ± 0.03	−	−	−
	1‐Octen−3‐ol	1441.00	747.72	−	0.96 ± 0.02	5.16 ± 0.05	−
	1‐Heptanol	1446.90	752.34	10.82 ± 0.06	−	5.46 ± 0.02	1.83 ± 0.03
	2‐Ethyl−1‐hexanol	1480.40	778.72	0.55 ± 0.03	0.50 ± 0.01	0.17 ± 0.04	0.61 ± 0.03
	(E)−2‐Hepten−1‐ol	1502.80	796.27	−	0.81 ± 0.02	0.17 ± 0.01	−
	2‐Nonanol	1508.20	800.22	−	0.63 ± 0.01	−	0.17 ± 0.02
	Linalool	1538.70	822.73	−	0.75 ± 0.01	−	0.97 ± 0.02
	1‐Octanol	1548.90	830.21	2.93 ± 0.03	1.09 ± 0.01	2.18 ± 0.03	2.73 ± 0.01
	(E)−2‐Octen−1‐ol	1604.70	871.20	1.16 ± 0.02	0.60 ± 0.01	1.50 ± 0.03	0.52 ± 0.01
	2‐Furanmethanol	1659.40	909.25	0.22 ± 0.01	0.41 ± 0.01	−	0.03 ± 0.01
	(E)−2‐Nonen−1‐ol	1706.10	941.51	1.33 ± 0.02	0.60 ± 0.02	0.35 ± 0.01	0.67 ± 0.02
	1‐Decanol	1753.40	972.74	0.27 ± 0.02	0.20 ± 0.01	−	−
	Benzenemethanol	1884.00	1056.50	0.14 ± 0.01	0.19 ± 0.01	−	0.09 ± 0.01
	Benzeneethanol	1920.70	1079.09	0.06 ± 0.01	0.20 ± 0.02	0.04 ± 0.01	0.04 ± 0.01
	2,4‐Decadien−1‐ol	1990.50	1121.27	0.46 ± 0.01	0.45 ± 0.02	0.87 ± 0.01	−
Aldehydes	Acetaldehyde	833.60	226.07	−	0.07 ± 0.01		−
	1‐Octanal	1286.60	618.70	0.27 ± 0.02	0.22 ± 0.03	0.18 ± 0.01	0.16 ± 0.02
	Nonanal	1391.80	708.58	1.19 ± 0.02	0.98 ± 0.01	0.58 ± 0.03	1.11 ± 0.02
	Benzaldehyde	1538.30	822.40	0.32 ± 0.01	−	−	−
Esters	Butanoic acid, methyl ester	990.00	336.22	0.08 ± 0.01	0.11 ± 0.02	0.03 ± 0.01	0.12 ± 0.01
	Butanoic acid, ethyl ester	1038.10	381.65	−	−	−	0.25 ± 0.01
	Hexanoic acid, ethyl ester	1230.00	567.04	−	−	−	1.67 ± 0.01
	Acetic acid, hexyl ester	1270.50	604.04	−	−	0.03 ± 0.01	0.08 ± 0.01
	Octadecanoic acid, methyl ester	2216.40	1248.78	0.09 ± 0.01	0.30 ± 0.02	0.07 ± 0.01	0.06 ± 0.01
	Hexadecanoic acid, ethyl ester	2252.90	1268.27	0.14 ± 0.02	0.27 ± 0.01	0.11 ± 0.03	0.55 ± 0.01
	1,2‐Benzenedicarboxylic acid, dibutyl ester	2547.80	1450.55	0.37 ± 0.01	2.13 ± 0.04	0.20 ± 0.02	21.00 ± 0.11
Ketones	5‐Methyl−2‐hexanone	1180.80	520.92	0.74 ± 0.01	−	−	−
	2‐Heptanone	1182.70	522.77	−	0.39 ± 0.02	0.12 ± 0.01	−
	2‐Octanone	1283.40	615.76	0.25 ± 0.01	−	−	−
	1‐Octen−3‐one	1299.90	630.88	0.24 ± 0.02	−	−	−
	2‐Nonanone	1390.00	706.98	−	0.11 ± 0.01	−	−
	2,3‐Octanedione	1640.70	896.26	−	−	0.02 ± 0.01	−
	1‐Phenyl‐ethanone	1667.50	914.88	0.16 ± 0.01	−	0.09 ± 0.01	0.24 ± 0.02
	β‐Damascenone	1834.10	1025.09	−	0.28 ± 0.03	−	−
Others	2‐Pentyl‐furan	1221.30	559.06	13.30 ± 0.02	0.24 ± 0.01	0.61 ± 0.02	0.57 ± 0.01
	2‐Acetylthiazole	1661.40	910.60	0.13 ± 0.01	0.27 ± 0.02	0.07 ± 0.01	0.28 ± 0.02
	Benzothiazole	1984.10	1117.39	0.06 ± 0.02	0.08 ± 0.01	0.03 ± 0.01	0.09 ± 0.01

Note

Results are expressed as mean ±standard deviation (*n* = 3).

Abbreviations: RI, retention indices; RT, retention time.

Acid is an important component produced in the sugar metabolism by *Lactobacillus* (de la Fuente et al., 2021). Starches in cereals are decomposed by enzymes into fermentable sugars, which are then metabolized by microorganisms into corresponding acids (Sripriya et al., 1997). We analyzed the acids in all samples; acetic acid, hexanoic acid, octanoic acid, and nonanoic acid were detected in all samples. Among these detected acidic compounds, acetic acid and hexanoic acid were the two main acids. The highest acetic acid content was observed in quinoa, which might be related to the content of fermentable sugars in quinoa enzymatic hydrolysate. Hexanoic acid, octanoic acid, and nonanoic acid are produced during lipid degradation or carbohydrate metabolism (Goswami et al., 2018). Linoleic acid is the main starting material for the formation of hexanal (Lee et al., 2016). During fermentation, hexanal is oxidized to hexanoic acid. Previous study has shown that millets are rich in linoleic acid (Li et al., 2021), which may be the reason for the higher content of hexanoic acid in millets.

Alcohols are the end products in the metabolism of glucose and amino acids by microorganisms (Cheng, 2010) and endow an elegant aroma and a soft taste with final fermentation products. The results showed that hexanol, octanol, and heptanol were produced during the fermentation process with *L*. *reuteri*. Hexanol and heptanol are important flavor compounds in fermented dairy products (Dan et al., 2017). Aldehydes can be reduced to alcohols or oxidized to acids under the action of microorganisms (Yi et al., 2021). Thus, hexanol might be produced by the reduction of hexanal. Hexanol was the main alcohol of coix seed and millet after fermentation, which may be related to the linoleic acid content in millet and coix seed. While ethanol was the main alcohol of quinoa after fermentation, less ethanol was detected in brown rice. This may be related to the differences in the composition of sugars in different substrates. It is reported that the presence of β‐fructofuranosidase in *L. reuteri* can hydrolyze sucrose to glucose and fructose (Cuezzo De Ginés, 2000). Glucose and fructose are metabolized by different pathways. Glucose was fermented to produce lactic acid, acetic acid, and ethanol by the phosphoketolase pathway. Fructose was reduced to mannitol as an electron acceptor, thereby producing the cofactor NAD^+^, and converting acetyl phosphate to acetic acid instead of ethanol (Gerez et al., 2008). In this work, the fermentable sugars formed by enzymolysis of different grains may be greatly different, thereby resulting in the difference of ethanol content after fermentation. However, further experiments are needed to confirm. In addition, benzenemethanol, linalool, benzeneethanol, 2‐furanmethanol, and citronellol were detected. Although the contents of these alcohols were low, they endowed the products with a distinctive aroma (Vilanova et al., 2009).

Aldehydes are not only important flavor components, but also the precursors of many compounds, such as acids and alcohols (Zhao et al., 2018). Octanal and nonanal were detected in all samples after the fermentation process. Octanal has a strong fruity aroma and nonanal has a strong oily and sweet orange aroma (Xu et al., 2019). Furthermore, 3‐furaldehyde and benzaldehyde were also detected in coix seed substrate. 3‐Furfuraldehyde is an important precursor of 2‐furanmethanol and tetrahydrofuran. Benzaldehyde is an aromatic compound with a bitter almond flavor and a fruit flavor (Pongsetkul et al., 2017). Furfural and acetaldehyde were detected in the quinoa substrate.

Esters are also important flavor components. During the fermentation process, lactic acid bacteria produce esters and produce a special aroma with the products (Wang et al., 2020). Butanoic acidmethyl ester with the aromas of apple and cheese was detected in these cereal substrates (Zhao, Wang, et al., 2021). Butanoic acid ethyl ester and hexanoic acid ethyl ester were also detected in brown rice. In addition, we also detected octanoic acid methyl ester and acetic acidhexyl ester. Esters are generally characterized by fruity aroma and regarded as the crucial volatile compounds due to their low threshold values (Rita et al., 2011).

The ketone compounds in four cereal substrates showed significant differences. 5‐Methyl‐2‐hexanone was the main ketone compound in the coix seed substrate with a pleasant aroma. In addition, aromatic compounds such as 2‐octenone and 1‐octene‐3‐ketone were detected in coix seed. 2‐Heptanone and beta‐damascenone were the main ketone components in quinoa. 2‐Heptanone has a fruity aroma and β‐damascenone has a strong rose fragrance (Carneiro et al., 2006; Yan et al., 2020). De Schutter et al. (2008) pointed out that lower pH is beneficial to the release of β‐damascenone. Therefore, the decrease of pH during the fermentation process contributes to the formation of β‐damascenone. 2‐Heptanone and 2,3‐Octanedione were also detected in the millet substrate.

Furan and thiazole are the main heterocyclic compounds. 2‐Pentylfuran, 2‐acetylthiazole, and benzothiazole were detected in four substrates. 2‐Pentylfuran is the most abundant heterocyclic compound with the aroma characteristics of vegetables and fruits and can be produced by the oxidative degradation of unsaturated fatty acids (Min et al., 2003; Tang et al., 2021). Yin et al. (2020) indicated that the content of 2‐pentylfuran was decreased from 23% to 10% in coix seed fermented with *Lactobacillus plantarum*. The content of 2‐pentylfuran detected in coix seed substrate was similar to previous results. In addition to the heterocyclic compounds mentioned earlier, pyridine and pyrazine usually generated by the Maillard reaction or amino acid decomposition were also detected in fermented substrates (Zhou et al., 2021). In general, the substrate difference was mainly responsible for the overall flavor difference. During the fermentation process, microorganisms metabolized different substrates to produce different aromatic components. In our study, *L*. *reuteri* showed different aromatic characteristics in four cereal substrates. After the fermentation process, alcohols and acids are dominant flavor components. Aldehyde, ketone, and ester compounds contributed little to the flavor, but the interaction between alcohol, aldehyde, and ketone produced a harmonious and delicate aroma, which had a positive influence on the flavor of products (Zhang et al., 2019).

### 3.5 Taste characteristics by e‐tongue assessment

**FIGURE 4:**
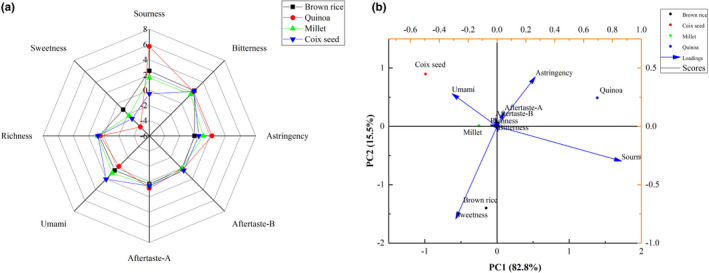
Radar charts (a) and principal component analysis (PCA) plot (b) about the taste of the beverage fermented by *Limosilactobacillus reuteri*

In recent years, electronic noses and electronic tongues have been widely used in the analysis of tastes (Chen et al., 2021). Electronic tongues convert electrical signals into relevant taste signals so as to distinguish the taste of foods and can objectively evaluate the taste differences among foods (Lao et al., 2020). We analyzed the taste characteristics of four substrates after the fermentation process. Bitterness, aftertaste A, aftertaste B, or richness showed no significant difference among the four cereal substrates after fermentation (Figure 4). Sour, umami, and sweet showed significant differences among the four cereal substrates. Quinoa had the most obvious sour taste due to more acids produced in the fermentation process, as confirmed in the above pH measurement results and organic acids. In addition, the astringency of the fermented quinoa sample was the most obvious due to the large quantity of saponins in quinoa (Suárez‐Estrella et al., 2021). Coix seed had the most obvious umami flavor after the fermentation process, which may be the produce of more umami components, such as glutamate and nucleotide (Vinther Schmidt et al., 2021). In the study, we did not find a large quantity of free glutamate (data not shown), indicating that the umami flavor in coix seed was ascribed to nucleotide. Another work of ours also confirmed this result (Yang et al., 2022).

## 4 CONCLUSIONS

In conclusion, our research clearly showed the potential of functional cereals in developing probiotic cereal beverages. Although fermented dairy products are considered as the classic carrier of probiotics, nondairy products of probiotics also receive great attention. In this work, we prepared several probiotic beverages using cereal enzymatic hydrolysates. According to our results, cereal enzymatic hydrolysates are ideal substrates for probiotics growth. Therefore, it would be used as a new probiotics carrier to produce cereal beverages with high viable count. In addition, probiotics fermentation can improve the flavor and enhance the sensory of the beverage. However, further studies on some of the changes during storage and the health benefits after consumption, as well as improving the beverage acceptability to consumers, should be conducted.

## CONFLICT OF INTEREST

The authors declare no financial or commercial conflict of interest.

## AUTHOR CONTRIBUTION

**Zhoujie Yang**: Data curation, formal analysis, investigation, methodology, visualization, and writing – original draft. **Xiaoli Zhu**: Investigation and methodology. **Anyan Wen**: Writing – review and editing and supervision. **Likang Qin**: Conceptualization, writing – review and editing, funding acquisition, and supervision.

## ETHICAL APPROVAL

This study does not involve any human or animal testing.

## Data Availability

The data that support the findings of this work are available on request from the authors.
